# Virological Breakthrough: A Risk Factor for Loss to Followup in a Large Community-Based Cohort on Antiretroviral Therapy

**DOI:** 10.1155/2011/469127

**Published:** 2011-05-08

**Authors:** Catherine Orrell, Richard Kaplan, Robin Wood, Linda-Gail Bekker

**Affiliations:** Desmond Tutu HIV Centre, Institute of Infectious Disease and Molecular Medicine and Department of Medicine, University of Cape Town, Cape Town 7925, South Africa

## Abstract

*Background*. We have previously shown that 75% of individuals on antiretroviral therapy (ART) in a resource-limited setting who experienced virological breakthrough to >1000 copies/mL were resuppressed after an intensive adherence intervention. This study examines the long-term outcomes of this group in order to understand the impact of the adherence intervention over time. *Methods*. ART-naïve adults commencing ART between September 2002 and December 2009 were reviewed. Those who achieved suppression (<50 copies/mL) were categorised by subsequent viral load: any >1000 copies/mL (virological breakthrough) or not. Those with breakthrough were sub-categorised by following viral load into failed (VL > 1000 copies/mL) or resuppressed (VL < 1000 copies/mL). Their outcome (lost-to follow-up, death, in care on first-line therapy or in care on second-line therapy) was determined as of the 13th April 2010. *Findings*. 4047 ART-naïve adults commenced ART. 3086 had >2 viral loads and were included in the analysis. 2959 achieved virological suppression (96%). Thereafter 2109 (71%) remained suppressed and 850 (29%) experienced breakthrough (*n* = 283 (33%) failed and *n* = 567 (67%) resuppressed). Individuals with breakthrough were younger (*P* < .001), had lower CD4 counts (*P* < .001), and higher viral loads (*P* < .001) than those who remained suppressed. By 7 years the risk of breakthrough was 42% and of failure 15%. Fewer adults with breakthrough remain in care over time (*P* < .001). Loss to care is similar whether the individuals failed or resuppressed. *Interpretation*. While 67% of those who experience initial virological breakthrough resuppress after an adherence intervention, these individuals are significantly less likely be retained in care than those who remain virologically suppressed throughout.

## 1. Introduction

Adherence to antiretroviral therapy is the key to successful treatment outcomes at both individual and programmatic levels. It has been shown that individuals taking 95% or more of their medication will maintain virological suppression and that this is readily achieved in resource-limited settings [1–3]. More recent data from larger nonnucleoside-based ART regimens show that these regimens allow for lower levels of adherence after initial viral suppression, perhaps >80%, before virological breakthrough occurs [4, 5].

Poor ART adherence can be assessed by the use of a selection of objective or subjective adherence measures. These include 3-day recall, a visual analogue scale [6], and a count of tablet returns or pharmacy refills [7]. Many ART programmes in resource-limited settings have had the opportunity to implement adherence-focused systems from the outset and have shown exceptionally high proportions of individuals with virological suppression at any visit [8–10].

If poor adherence is persistent or is not detected, virological breakthrough may occur. At this point, intensive adherence support is required to initiate a change in tablet-taking behaviour which can result in resuppression of the HIV before resistance develops. In 2007, this group showed that 75% of individuals with virological breakthrough to >1000 copies/mL resuppressed to <50 copies/mL at the subsequent viral load after an intensive adherence intervention [10]. The impact of this increase in viral load on the longer term course of ART is unknown. There is a possibility that the period of poor adherence with subsequent inadequate ART drug levels may have allowed the HIV to generate resistance mutations resulting in earlier failure than observed in those with no episodes of virological breakthrough. 

In the previously published study, numbers of naive adults on treatment were limited (*n* = 929) and outcomes could only be determined to 32 months.

The objective of this study was to examine the long term programmatic outcomes of individuals who had previously experienced virological breakthrough to >1000 copies/mL and then resuppressed. We aimed to determine whether the impact of the intervention was longstanding or whether it resulted in differing outcomes including increased rates of virological failure and loss to followup/retention in care, compared to those with continued virological suppression.

## 2. Methods

### 2.1. Site Description

Antiretroviral delivery at the Hannan Crusaid Treatment Centre (HCTC) has been described previously [10]. The HCTC is one of the Department of Health ART sites in the Nyanga district, a township near Cape Town, South Africa. An estimated 300,000 people live in this district, which had an antenatal HIV-1 seroprevalence rate of 27.9% in 2008 [11]. The clinic opened in September 2002 and by December 2009 cared for 2991 adults and 270 children needing ART. Antiretroviral therapy and associated monitoring costs are provided free of charge in South Africa. HIV RNA load and CD4 cell count assessments were completed pretreatment and every 16 weeks while taking ART. Viral load assays were done using the branch DNA hybridisation technique (Bayer HIV-1 RNA 3.0 assay (branch DNA)). Additional safety tests were performed according to the 2004 edition of the national ART protocol and varied according to the treatment regimen [12].

### 2.2. Drug Regimens

Until the 2010 edition of the South African National Antiretroviral guidelines, first-line ART included stavudine (d4T), lamivudine (3TC), and either efavirenz or nevirapine. Zidovudine (AZT) or tenofovir could be substituted for d4T-related toxicity. ART for second-line therapy included AZT, Didanosine (ddI), and lopinavir/ritonavir [12, 13].

### 2.3. Adherence Support

The HCTC employed HIV-positive lay counselors to educate each individual prior to initiating ART and to provide on-treatment adherence support. Treatment preparedness included three small group education sessions and a home visit by a counselor assigned to the area where the individual lived. Further home visits occurred monthly after starting treatment and continued until viral suppression was achieved. Thereafter, home visits were only carried out if the individual was flagged as nonadherent, experienced virological breakthrough, or was identified as a defaulter through missed clinic visits.

### 2.4. Adherence Interventions

Patients on ART with a viral load >1000 copies/mL (virological breakthrough) at any followup visit received a targeted adherence intervention. These individuals were required to attend extracounselling sessions, which focussed on adherence issues, and weekly home visits were recommenced. The individual was issued with a pill box and a dosing diary. The viral load was repeated six to eight weeks after this intervention. Based on the second test, patients were either regarded as having resuppressed or having failed treatment. Patients with a second viral load >1000 copies/mL were prepared for second-line therapy while resuppression to a viral load <1000 copies/mL allowed for continuation on first-line therapy, with high-frequency home visits continuing. If the viral load had fallen to <50 copies/mL routine clinic care was recommenced.

### 2.5. Study Design and Analysis

Data were accessed from clinical (age, gender, WHO stage) and laboratory records (CD4 counts and viral load) that were maintained on all patients included in the HCTC ART programme. These records were transferred on a weekly basis to an off-site database. Outcomes which included death, loss to followup, or transfer out were noted in the database and the date of the outcome was captured.

### 2.6. Definitions

“Virological breakthrough” refers to any individual whose viral load reached >1000 copies/mL after previous suppression to <50 copies/mL. “Resuppressed” refers to those individuals whose viral load subsequent to the one at virological breakthrough was <1000 copies/mL. “Virological failure” refers to individuals with two consecutive viral loads >1000 copes/mL. “Retention in care” refers to individuals still receiving care either on-site or at another site (transfer out). “Losses to care” included deaths and individuals lost to followup.

### 2.7. Treatment Cohort

Patients commencing ART at the clinic between 2 September 2002 and 31 December 2009 were reviewed retrospectively. Those <15 years of age and those who were nonnaïve at entry to the programme were excluded. Also excluded were those patients who had 1 or no viral load after commencement of treatment as well as those who never achieved virological suppression on ART. All individuals who achieved suppression after ART commencement and later experienced virological breakthrough were analysed and divided into those who went on to virological failure and those who resuppressed and continued on first line. Long-term outcomes including death, transfer out to care in another service, or loss to followup were determined for all individuals in the cohort.

### 2.8. Statistical Analysis

Demographic and baseline data were described using medians and proportions as appropriate. Baseline characteristics were compared using non-parametric statistics for data not normally distributed.

Patients were right censored on 13 April 2010 if they remained in care. Patients who were more than 12 weeks late for a scheduled visit were considered lost to followup, and their last visit to the clinic was used as last date in care. Patients who were transferred out were considered to still be in care at the time of censoring. Kaplan-Meier survival analysis was used to assess risks of failure and loss to care.

### 2.9. Ethical Review

The University of Cape Town Research Ethics Committee approved data capture from the ART site at the HCTC. All individuals who enrolled onto the programme provided written informed consent.

## 3. Results

**Figure 1:**
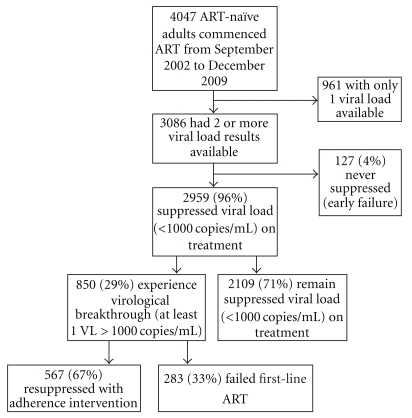
A flow diagram describing the cohort with virological breakthrough (>1000 copies/mL) as a subset of ART-naïve adults commencing treatment at the HCTC and subsequent outcomes.

As of the 31st December 2009, 4967 individuals had commenced ART at the HCTC. After excluding 375 children under the age of 15 years and 545 adult individuals who were transferred in on ART from another site, 4047 ART-naïve adults were available for review (Figure 1). A further 961 people were excluded from the analysis since they had had fewer than two viral loads on treatment, either as they died early into treatment (*n* = 256, 27%), were lost to followup (*n* = 258, 27%), transferred to care elsewhere (*n* = 121, 13%), or were within the first eight months of treatment (*n* = 326, 33%). A small proportion of the cohort never suppressed on treatment and experienced early failure (*n* = 127, 4.1%). These individuals were also excluded (Figure 1). The demographics of the remaining 2959 individuals used for this analysis are noted in Table 1.

The majority of individuals had at least one point where they suppressed their viral load to <50 copies/mL (*n* = 2959, 96%). Of those that initially suppressed on treatment, the majority did not have a subsequent viral load >1000 copies/mL (*n* = 2109, 71%). The remaining 850 individuals (29%) experienced virological breakthrough in that they had at least one viral load >1000 copies/mL while on treatment (Figure 1). Those with virological breakthrough were significantly younger, had a lower baseline CD4 count (*P* = .0003), and a higher viral load (*P* = .002) than those who remained suppressed throughout (Table 1).

Of the 850 individuals who experienced virological breakthrough, 567 resuppressed and 283 failed virologically (Figure 1). There was no significant difference in the age or stage between the above groups at baseline; however, individuals who failed ART had significantly lower CD4 counts and higher viral loads at baseline than those who resuppressed. 

**Figure 2:**
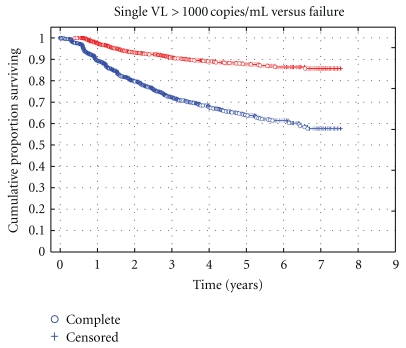
A Kaplan-Meier survival curve depicting risk of an initial virological breakthrough (first viral load >1000 copies/mL after initial suppression—lower curve) and subsequent risk of virological failure (second consecutive viral load >1000 copies/mL—upper curve). Of those with virological breakthrough an expected 66% will resuppress after an adherence intervention.

The median time to breakthrough was 1.39 years (IQR 0.75–2.46 years), and to failure, 1.51 years (IQR 0.99–2.64 years).  Figure 2 is a Kaplan-Meier survival curve showing the risk of virological breakthrough and subsequent failure for the whole cohort (*n* = 2959). At seven years into treatment, up to 42% of the population on ART have a risk of experiencing at least one viral load >1000 copies after prior suppression. Only 15% have a risk of going on to fail suggesting that 67% of those who experienced breakthrough might be expected to resuppress at the next viral load. 

Table 2 describes the long-term outcome of those who resuppressed. Of those who never experienced virological breakthrough 77% remained in care at the time of this study. There was some loss due to transfer out to another clinical setting (*n* = 186, 8.9%) and to death (*n* = 64, 3.1%). Eleven percent (*n* = 235) were lost to followup. The outcomes for those who had a single viral load >1000 copies/mL and who then resuppressed differ significantly, with fewer remaining in care (*n* = 348, 61%; *P* = .0000), largely as a function of increased loss to followup (*n* = 123, 23%; *P* = .0000). There was no significant difference in proportion transferred to care elsewhere (*n* = 62, 11%; *P* = .1623) or in deaths (*n* = 25, 4.4%; *P* = .1184).

While all individuals who never experienced virological breakthrough remain on first-line therapy, 46 individuals (8%) of those who resuppressed after breakthrough went on to fail at a time subsequent to their next consecutive viral load and switched to second-line therapy.

**Figure 3:**
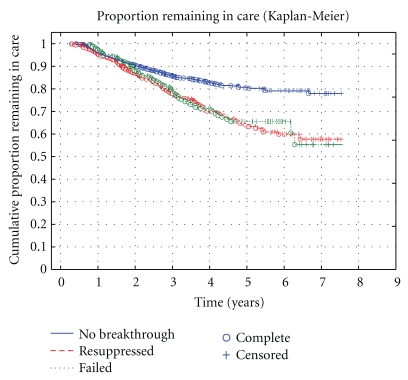
A Kaplan-Meier survival curve depicting risk of loss to care overtime. Losses include deaths and those lost to followup. Those who never experience virological breakthrough are more likely to remain in care overtime. Losses to care are greater in those who experience breakthrough and do not differ by future virological outcomes (failed or resuppressed).

Figure 3 is a Kaplan-Meier survival curve showing the risk of loss to care described above overtime. At seven years into care, it can be expected that 78% of those who remain suppressed throughout their ART remain in care. Losses are due to either death or loss to followup. The curves for those who experienced virological breakthrough do not differ as a function of resuppression or failure, with only 55–58% of these groups remaining in care by 7 years.

## 4. Discussion

The objective of this study was to determine whether those individuals on ART who experienced a single episode of virological breakthrough after prior successful suppression had similar long-term virological outcomes to individuals who remain suppressed on ART throughout. 

Of note, the majority of this treatment cohort achieved virological suppression (96%), a much higher proportion than noted in a recent systematic review of sub-Saharan African ART programmes where only 78% of treated patients achieved virological suppression at six months [14]. Seventy-one percent of this group continued to be suppressed at the censor date for this analysis. Of the 29% who experienced virological breakthrough, two-thirds resuppressed after the intensive adherence intervention: a similar proportion to that seen in 2005 [10]. However, the risk of experiencing virological breakthrough continued to increase overtime, so that by seven years in care there was a relatively high risk (42%) that an individual would have experienced breakthrough and a 15% risk of failure of first-line therapy. 

Preliminary data from 2005 showed that the risk of failure remained relatively low in individuals who had virological breakthrough [10]. This study, of an expanded cohort, suggests that these individuals have different outcomes from those who remained successfully suppressed. While 77% of those who remained suppressed throughout were retained in care seven years into the programme, this only applied to 58% of patients who experienced virological breakthrough. This is largely a function of increasing loss to followup overtime. These individuals were younger and more ill with lower CD4 counts and higher viral loads at the time of starting ART. It is of note that the risk of loss to followup for patients who have virological breakthrough and then resuppress is the same as for patients who fail treatment.

A systematic review of patient retention in ART programmes estimated rates across 33 cohorts to range from 24 to 77% at only two years into the programme. Death and loss to followup accounted for the majority of these losses, as in our programme [15]. 

Although the overall outcomes of our programme may seem reasonable compared to those of other current programmes, as the expansion of antiretroviral services continue, so must the effort to improve adherence to care. While our adherence intervention appears successful in that two-thirds of those who experience virological breakthrough did resuppress it is important to note that this intervention does not impact on loss to followup.

Previous studies have identified subgroups of patients who are at risk of LTFU. These include pregnant women and younger people on ART [16]. This study identified patients with virological breakthrough as another of these subgroups. It seems that those who struggle to adhere to therapy, initially noted by an increase in viral load, signaling less than ideal adherence to taking ART tablets, may also not adhere to the program. It should also be considered that the intensity of the adherence intervention itself, while successful for some individuals, may deter others from remaining in care. It is a weakness of this study that this is left to speculation. Qualitative information from individuals lost to followup from this programme would be required to answer this in detail.

Intensive adherence programmes may result in retained first-line therapy for those individuals who have an initial viral breakthrough but they do not of themselves retain people in care. Remaining on first-line therapy is an important goal not only in terms of cost saving but also in terms of preserving treatment options in resource-constrained settings. However, increasing attention is being drawn to retention to care in these settings in order to secure public health impact. Adherence interventions should be targeted to those who have an increased risk of loss to followup and need to be broadened to address issues related to loss to care as well as tablet-taking behavior.

## Figures and Tables

**Table 1:** 

	All naïve adults with initial viral suppression: *n* = 2959			Naïve adults with initial virological breakthrough: *n* = 850		
	Remain suppressed	Initial viral breakthrough		Resuppressed	Failed	
Total number	2109	850	—	567 (67%)	283 (33%)	—
Female gender: *n* (%)	1415 (67)	585 (68)	*P* = .7733*	386 (68)	200 (71)	*P* = .7419*
Age at treatment start: mean years (±SD)	35 (±8.7)	33 (±8.0)	*P* = .0000**	34 (±8.3)	32 (±7.5)	*P* = .7591**
WHO stage 1 or 2: *n* (%)	611 (29)	228 (27)	*P* = .5099*	159 (28)	67 (24)	*P* = .2981*
WHO stage 3 or 4: *n* (%)	1495 (71)	622 (73)	*P* = .7068*	406 (72)	216 (76)	*P* = .5663*
WHO stage unknown	3	0	—	0	0	—
Baseline CD4: median cells/mm^3^ (IQR)	117 (62−168)	110 (50−163)	*P* = .0003^#^	110 (57−171)	83 (34−138)	*P* = .0000^#^
Baseline viral load: median copies/mL (IQR)	4.82 (4.39−5.26)	4.94 (4.51−5.30)	*P* = .0002^#^	4.86 (4.48−5.24)	5.07 (4.70−5.48)	*P* = .0000^#^

*Chi-squared.

**T-test.

^#^Mann-Whitney *U* test.

**Table 2:** Programmatic outcome of those who resuppressed compared to those who never experienced virological breakthrough.

	Never had breakthrough (*n* = 2109)	Breakthrough and resuppressed (*n* = 567)	*P* value
Lost to followup: *n* (%)	235 (11)	132 (23)	*P* = .0000*
Died on treatment: *n* (%)	64 (3.1)	25 (4.4)	*P* = .1184*
Transfer out: *n* (%)	186 (8.9)	62 (11)	*P* = .1623*
Continue in care: *n* (%)			
(i) On first line	1624 (77)	302 (53)	*P* = .0000*
(ii) Failed first line	0 (0)	46 (8)	*P* = .0000*

*Chi-squared (df = 1).

## References

[1] Arnsten JH, Demas PA, Farzadegan H (2001). Antiretroviral therapy adherence and viral suppression in HIV-infected drug users: comparison of self-report and electronic monitoring. *Clinical Infectious Diseases*.

[2] Bangsberg DR, Hecht FM, Charlebois ED (2000). Adherence to protease inhibitors, HIV-1 viral load, and development of drug resistance in an indigent population. *AIDS*.

[3] Paterson DL, Swindells S, Mohr J (2000). Adherence to protease inhibitor therapy and outcomes in patients with HIV infection. *Annals of Internal Medicine*.

[4] Nachega JB, Hislop M, Dowdy DW, Chaisson RE, Regensberg L, Maartens G (2007). Adherence to nonnucleoside reverse transcriptase inhibitor-based HIV therapy and virologic outcomes. *Annals of Internal Medicine*.

[5] Rosenblum M, Deeks SG, van der Laan M, Bangsberg DR (2009). The risk of virologic failure decreases with duration of HIV suppression, at greater than 50% adherence to antiretroviral therapy. *PLoS ONE*.

[6] Oyugi JH, Byakika-Tusiime J, Charlebois ED (2004). Multiple validated measures of adherence indicate high levels of adherence to generic HIV antiretroviral therapy in a resource-limited setting. *Journal of Acquired Immune Deficiency Syndromes*.

[7] Nachega JB, Hislop M, Dowdy DW (2006). Adherence to highly active antiretroviral therapy assessed by pharmacy claims predicts survival in HIV-infected South African adults. *Journal of Acquired Immune Deficiency Syndromes*.

[8] Orrell C, Bangsberg DR, Badri M, Wood R (2003). Adherence is not a barrier to successful antiretroviral therapy in South Africa. *AIDS*.

[9] Mills EJ, Nachega JB, Buchan I (2006). Adherence to antiretroviral therapy in sub-Saharan Africa and North America: a meta-analysis. *Journal of the American Medical Association*.

[10] Orrell C, Harling G, Lawn SD (2007). Conservation of first-line antiretroviral treatment regimen where therapeutic options are limited. *Antiviral Therapy*.

[11] Department of Health (2009). *2008 National Antenatal Sentinel HIV and Syphilis Prevalence Survey*.

[12] (2004). *South African National Antiretroviral Treatment Guidelines*.

[13] National Department of Health SA (2010). *Clinical Guidelines for the Management of HIV & AIDS in Adults and Adolescents*.

[14] Barth RE, van der Loeff MFS, Schuurman R, Hoepelman AI, Wensing AM (2010). Virological follow-up of adult patients in antiretroviral treatment programmes in sub-Saharan Africa: a systematic review. *The Lancet Infectious Diseases*.

[15] Fox MP, Rosen S (2010). Patient retention in antiretroviral therapy programs up to three years on treatment in sub-Saharan Africa, 2007–2009: systematic review. *Tropical Medicine and International Health*.

[16] Kaplan R, Orrell C, Zwane E, Bekker L-G, Wood R (2008). Loss to follow-up and mortality among pregnant women referred to a community clinic for antiretroviral treatment. *AIDS*.

